# Supplementary material for: Metabolic mapping of the human solute carrier superfamily
Source: Mol Syst Biol. 2025 May 12;21(6):560–98. doi: 10.1038/s44320-025-00106-4 (PMC12130231; doi:10.1038/s44320-025-00106-4)

Appendix for "Metabolic mapping of the human solute carrier superfamily"
Wiedmer, Teoh et al.

Table of Content

Appendix Figure S1. Analysis of variance in differential omics profiles and detection frequency of significant metabolite changes. 2

Appendix Figure S2. Enrichment analysis reveals functional properties significantly enriched in transcriptomic clusters. 4

Appendix Figure S3. Coherence analysis identifies clusters representing potentially shared signatures in both metabolome and transcriptome profiles. 6

Appendix Figure S4. Transcriptomic cluster 17 members share a common differential gene expression signature related to osmolyte transport. 7

Appendix Figure S1. Analysis of variance in differential omics profiles and detection frequency of significant metabolite changes.

(A) Principal component analysis of RNA-seq differential analyses for 441 SLCs. Differential analysis was performed between Dox-induced and uninduced samples (two replicates per condition). Dots represent differential analyses and are colored according to the structural fold of the SLCs. **(B)** Principal component analysis of targeted metabolomics differential analyses for 378 SLCs. Differential analysis was performed between Dox-induced and uninduced samples (four replicates per condition). Dots represent differential analyses and are colored according to the structural fold of the SLCs. **(C)** Frequency of significant changes per metabolite across all profiled cell lines. Frequency was calculated for each metabolite as the number of differential analyses with significant change divided by total number of differential analyses (in most cases equal to number of cell lines in the data set, i.e. 378, unless differential analysis could not be performed for some cell lines due to e.g. poor measurement of the metabolite or its normalization internal standards). **(D)** Scatterplot showing the frequency of a metabolite's measurement in a biological sample above the measurement of the lowest calibration sample in the same batch (taken as indicator of robust detection), versus the frequency of significant changes for 189 metabolites in the metabolomics data set (padj <0.05).

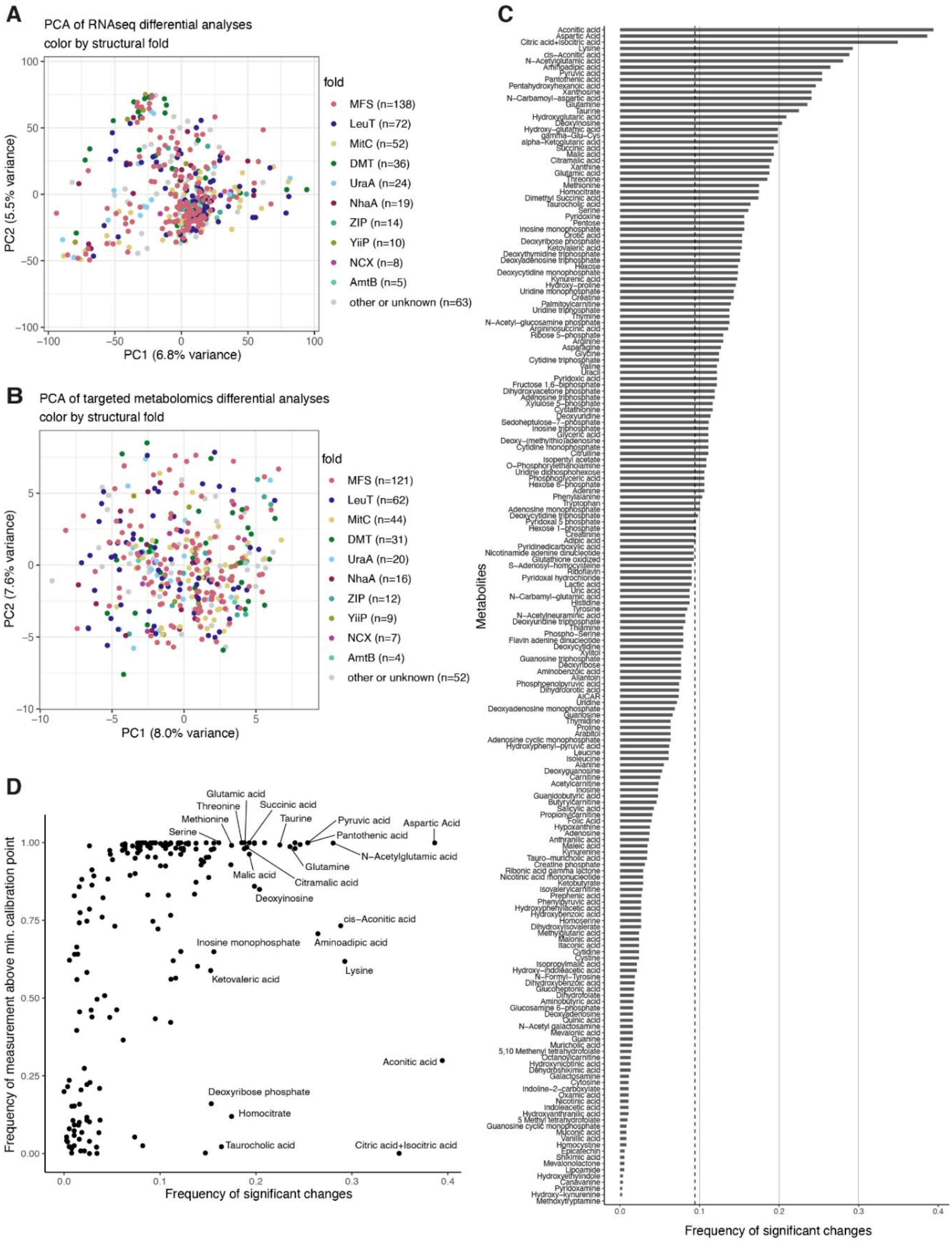
A
PCA of RNAseq differential analyses
color by structural fold
PC1 (6.8% variance)
PC2 (5.5% variance)
fold
MFS (n=138)
LeuT (n=72)
MitC (n=52)
DMT (n=36)
UraA (n=24)
NhaA (n=19)
ZIP (n=14)
YiiP (n=10)
NCX (n=8)
AmtB (n=5)
other or unknown (n=63)
B
PCA of targeted metabolomics differential analyses
color by structural fold
PC1 (8.0% variance)
PC2 (7.6% variance)
fold
MFS (n=121)
LeuT (n=62)
MitC (n=44)
DMT (n=31)
UraA (n=20)
NhaA (n=16)
ZIP (n=12)
YiiP (n=9)
NCX (n=7)
AmtB (n=4)
other or unknown (n=52)
C
Metabolites
Frequency of significant changes
D
Frequency of measurement above min. calibration point
Frequency of significant changes
Glutamic acid
Threonine
Succinic acid
Methionine
Taurine
Pyruvic acid
Aspartic Acid
Serine
Pantothenic acid
N-Acetylglutamic acid
Glutamine
Malic acid
Citramalic acid
Deoxyinosine
cis-Aconitic acid
Inosine monophosphate
Aminoadipic acid
Lysine
Ketovaleric acid
Aconitic acid
Deoxyribose phosphate
Homocitrate
Taurocholic acid
Citric acid+Isocitric acid

Appendix Figure S2. Enrichment analysis reveals functional properties significantly enriched in transcriptomic clusters.

(A) Colored horizontal bars mark SLCs with different functional properties as described in accompanying manuscript (Goldmann et al, 2024). Solid colors indicate significant enrichment (Fisher's test $p < 0.2$) of a property in the corresponding cluster(s), while faded colors denote SLCs in clusters where a property was not significantly enriched. Only functional properties significantly enriched in at least one cluster are shown.

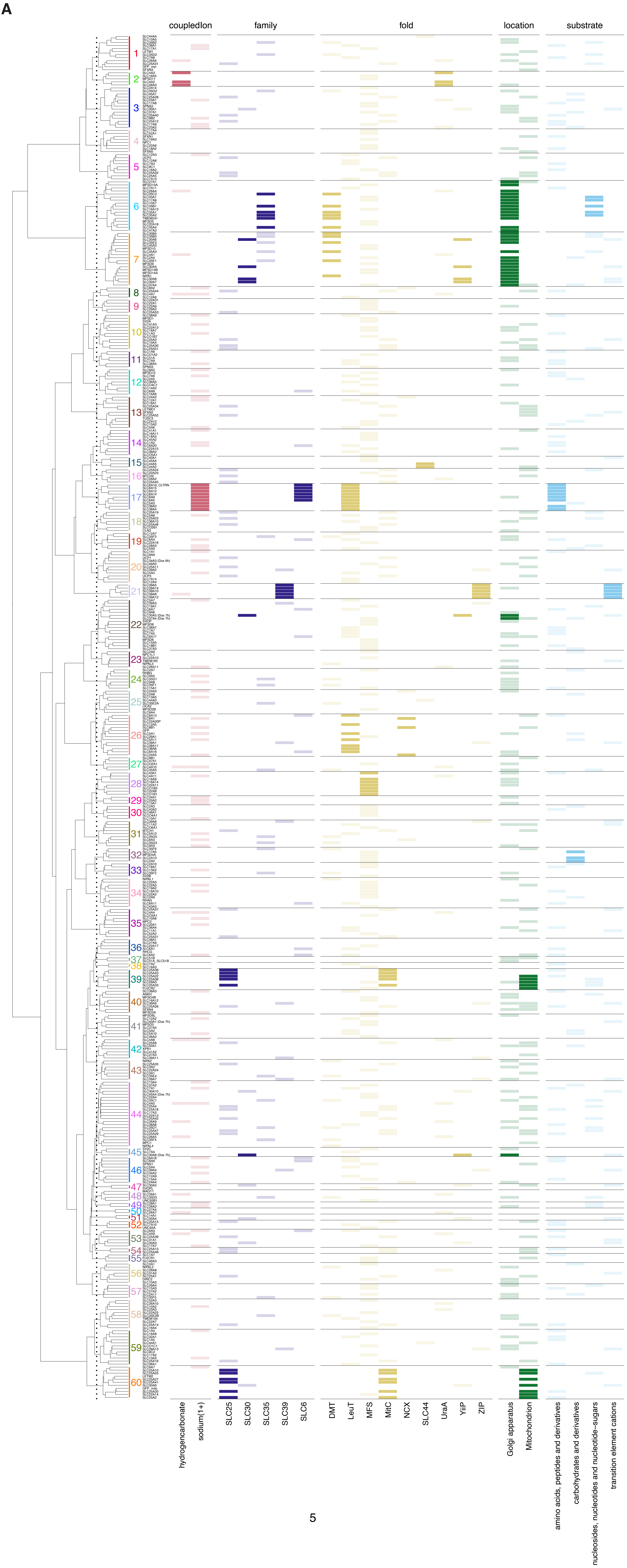
A
coupledIon
family
fold
location
substrate
1
2
3
4
5
6
7
8
9
10
11
12
13
14
15
16
17
18
19
20
21
22
23
24
25
26
27
28
29
30
31
32
33
34
35
36
37
38
39
40
41
42
43
44
45
46
47
48
49
50
51
52
53
54
55
56
57
58
59
60
hydrogencarbonate
sodium(1+)
SLC25
SLC30
SLC35
SLC39
SLC6
DMT
LeuT
MFS
MitC
NCX
SLC44
UraA
YiiP
ZIP
Golgi apparatus
Mitochondrion
amino acids, peptides and derivatives
carbohydrates and derivatives
nucleosides, nucleotides and nucleotide−sugars
transition element cations

Appendix Figure S3. Coherence analysis identifies clusters representing potentially shared signatures in both metabolome and transcriptome profiles.

(A) Contributions of individual clusters to mutual information (MI) between metabolomics and transcriptomics clustering. Clusters showing high MI contribute to the overall *similarity* between the clusterings. **(B)** Contributions of individual clusters to normalized variation of information (NVI) between metabolomics and transcriptomics clustering. Clusters showing relatively low NVI contribute to a low overall *distance* between the clusterings.

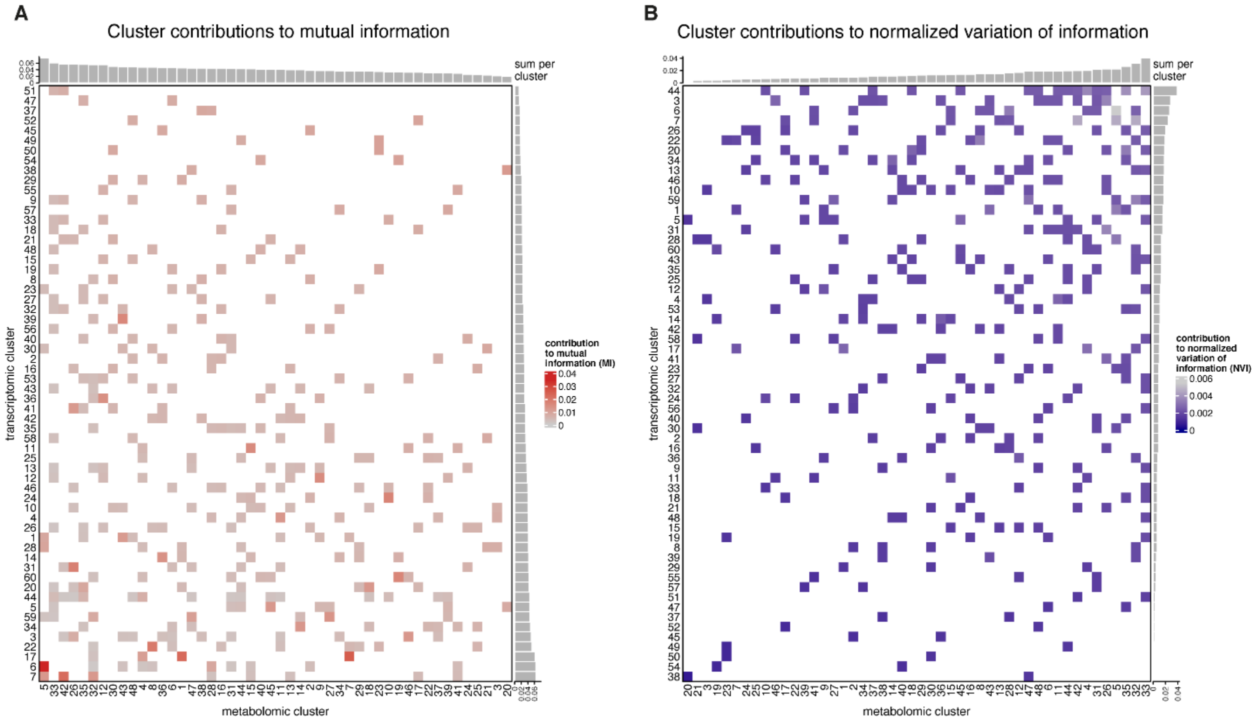

Appendix Figure S4. Transcriptomic cluster 17 members share a common differential gene expression signature related to osmolyte transport.

(A) Heatmap showing doxycycline (Dox) vs uninduced log2 fold changes of the 25 most differentially expressed genes in cluster 17 for each cluster member. Genes are indicated on x-axis and the clustered cell lines on the y-axis.

A

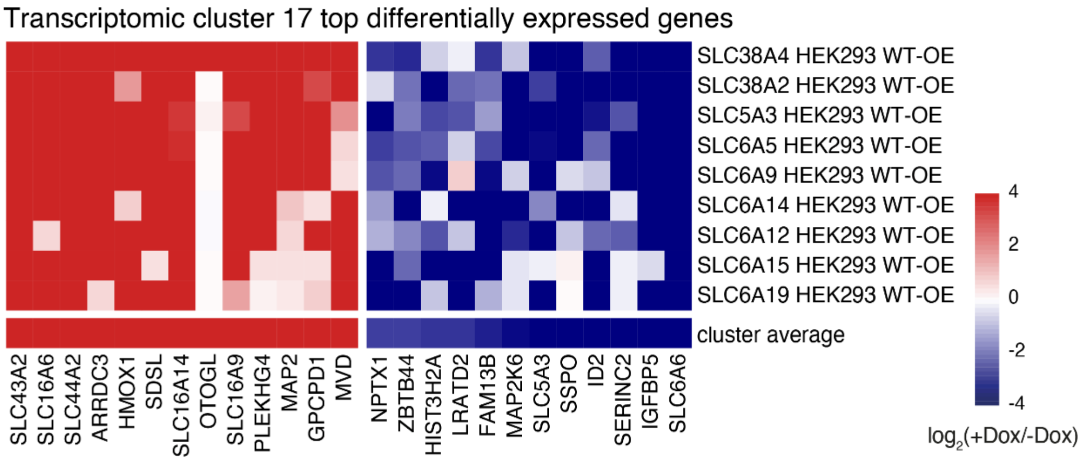

Supplement: Supplementary file 1 — Appendix [file 44320_2025_106_MOESM1_ESM.pdf]
