# Supplementary material for: Metabolic mapping of the human solute carrier superfamily
Source: Mol Syst Biol. 2025 May 12;21(6):560–98. doi: 10.1038/s44320-025-00106-4 (PMC12130231; doi:10.1038/s44320-025-00106-4)
Supplement: Supplementary file 15 — Expanded View Figures [file 44320_2025_106_MOESM15_ESM.pdf]

Expanded View Figures

Figure EV1. Comprehensive multi-omic coverage of the SLC superfamily.

(**A**) Heatmap illustrating coverage of 447 SLCs across five cancer cell lines considered for KO and KO-OE (HCT 116, LS180, 1321N1, SK-MEL-28, Huh-7) as well as Jump-In™ T-REx™ HEK293. Tpm > 1 was used as threshold for expression. (**B**) Number of SLCs with targeted metabolomics and transcriptomics data sets and respective parental cell lines included in this study. (**C**) Coverage of transcriptomics data sets in this study compared to the SLC superfamily (the RESOLUTE list of 447 SLCs). The coverage is given both in absolute numbers per family (top) and percentage of members per family (bottom). (**D**) Coverage of targeted metabolomics data sets in this study compared to the SLC superfamily (the RESOLUTE list of 447 SLCs). The coverage is given both in absolute numbers per family (top) and percentage of members per family (bottom). (**E**) Principal component analysis of 378 targeted metabolomics differential analyses. Differential analysis was performed between +Dox/−Dox samples. Data points are colored according to the parental cell lines. Cell line models and number of analyses per cell line are given in brackets. (**F**) Comparison of the average $\log_2$ fold change and frequency of significant changes ($P_{adj.} < 0.05$) for each metabolite across all differential analyses performed (378 SLCs). Metabolite data points are shaded according to the frequency of detection above the calibration minimum (0–1). (**G**) Profiled SLCs by targeted metabolomics and divided according to the substrate class of their annotated substrates. Colored bars indicate proportion of SLCs with significant changes upon differential analysis of metabolite abundance +/− doxycycline (Dox) induction, while gray bars indicate proportion of SLCs without significant metabolite changes. (**H**) Frequency of significant changes involving annotated substrates and metabolic conversions compared to frequency of all significant changes. All pairs of SLCs and targeted metabolites were grouped by a potential match of the SLC's annotated substrates to the targeted metabolite. The frequency of significant change was calculated per group and was found to be significantly higher for the 308 cases where a targeted metabolite could be directly matched to an annotated substrate for the overexpressed SLC. A smaller but also significant effect was found for the 2318 cases where a targeted metabolite could be matched via metabolic conversion to an annotated substrate for the overexpressed SLC (both Fisher's exact tests; error bars are the 95% confidence region of the perturbation frequency as calculated from the Fisher's test odds ratio estimate).

▶

 © The Author(s)

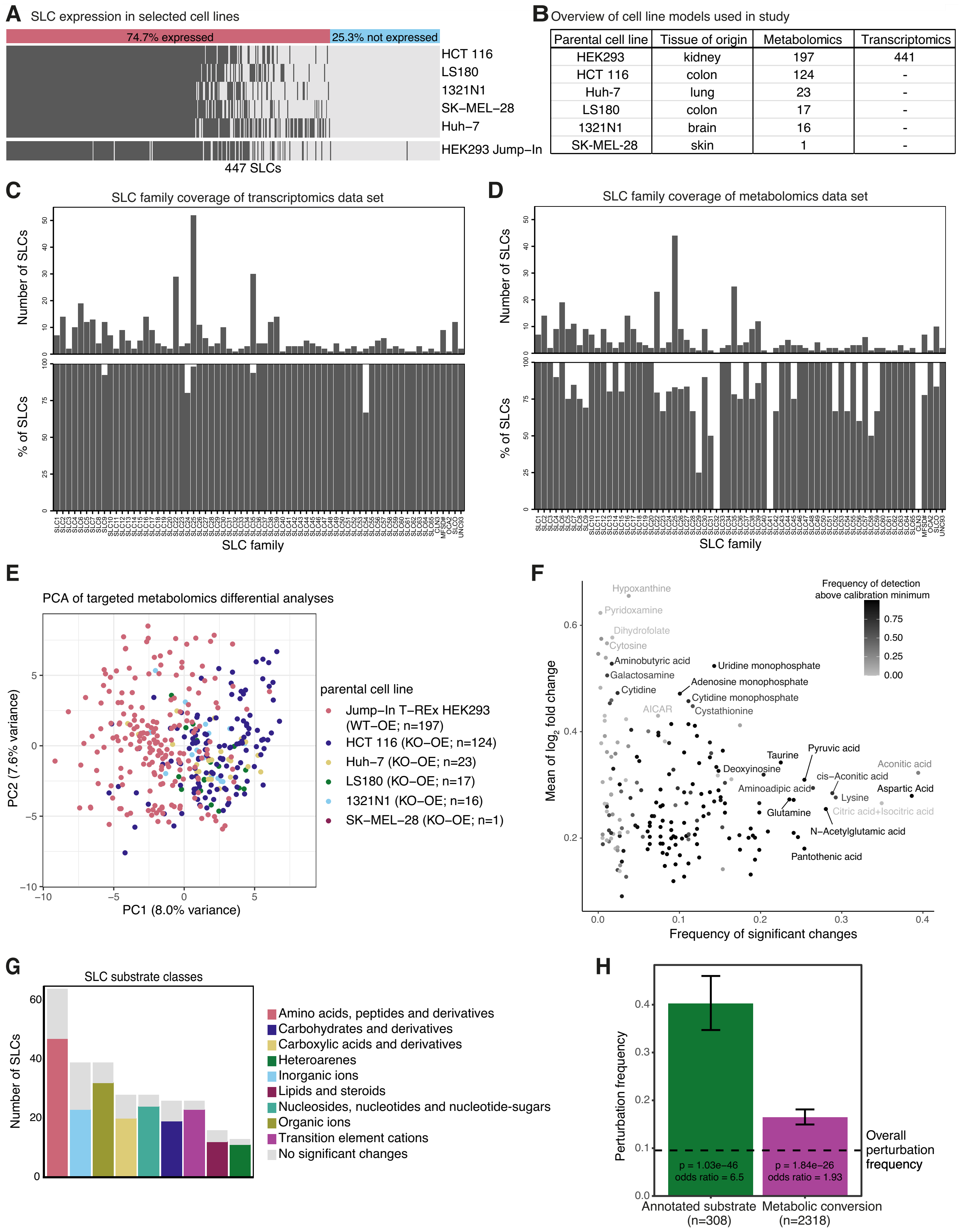
A SLC expression in selected cell lines
74.7% expressed
25.3% not expressed
HCT 116
LS180
1321N1
SK–MEL–28
Huh–7
HEK293 Jump–In
447 SLCs
B Overview of cell line models used in study
Parental cell line | Tissue of origin | Metabolomics | Transcriptomics
HEK293 | kidney | 197 | 441
HCT 116 | colon | 124 | -
Huh-7 | lung | 23 | -
LS180 | colon | 17 | -
1321N1 | brain | 16 | -
SK-MEL-28 | skin | 1 | -
C SLC family coverage of transcriptomics data set
Number of SLCs
% of SLCs
SLC family
D SLC family coverage of metabolomics data set
Number of SLCs
% of SLCs
SLC family
E PCA of targeted metabolomics differential analyses
PC2 (7.6% variance)
PC1 (8.0% variance)
parental cell line
Jump–In T–REx HEK293 (WT–OE; n=197)
HCT 116 (KO–OE; n=124)
Huh–7 (KO–OE; n=23)
LS180 (KO–OE; n=17)
1321N1 (KO–OE; n=16)
SK–MEL–28 (KO–OE; n=1)
F
Frequency of detection above calibration minimum
Mean of log2 fold change
Frequency of significant changes
Hypoxanthine
Pyridoxamine
Dihydrofolate
Cytosine
Aminobutyric acid
Galactosamine
Cytidine
Uridine monophosphate
Adenosine monophosphate
Cytidine monophosphate
Cystathionine
AICAR
Taurine
Pyruvic acid
Deoxyinosine
Aminoadipic acid
cis–Aconitic acid
Aconitic acid
Aspartic Acid
Lysine
Glutamine
Citric acid+Isocitric acid
N–Acetylglutamic acid
Pantothenic acid
G SLC substrate classes
Number of SLCs
Amino acids, peptides and derivatives
Carbohydrates and derivatives
Carboxylic acids and derivatives
Heteroarenes
Inorganic ions
Lipids and steroids
Nucleosides, nucleotides and nucleotide-sugars
Organic ions
Transition element cations
No significant changes
H
Perturbation frequency
p = 1.03e−46
odds ratio = 6.5
p = 1.84e−26
odds ratio = 1.93
Overall perturbation frequency
Annotated substrate (n=308)
Metabolic conversion (n=2318)

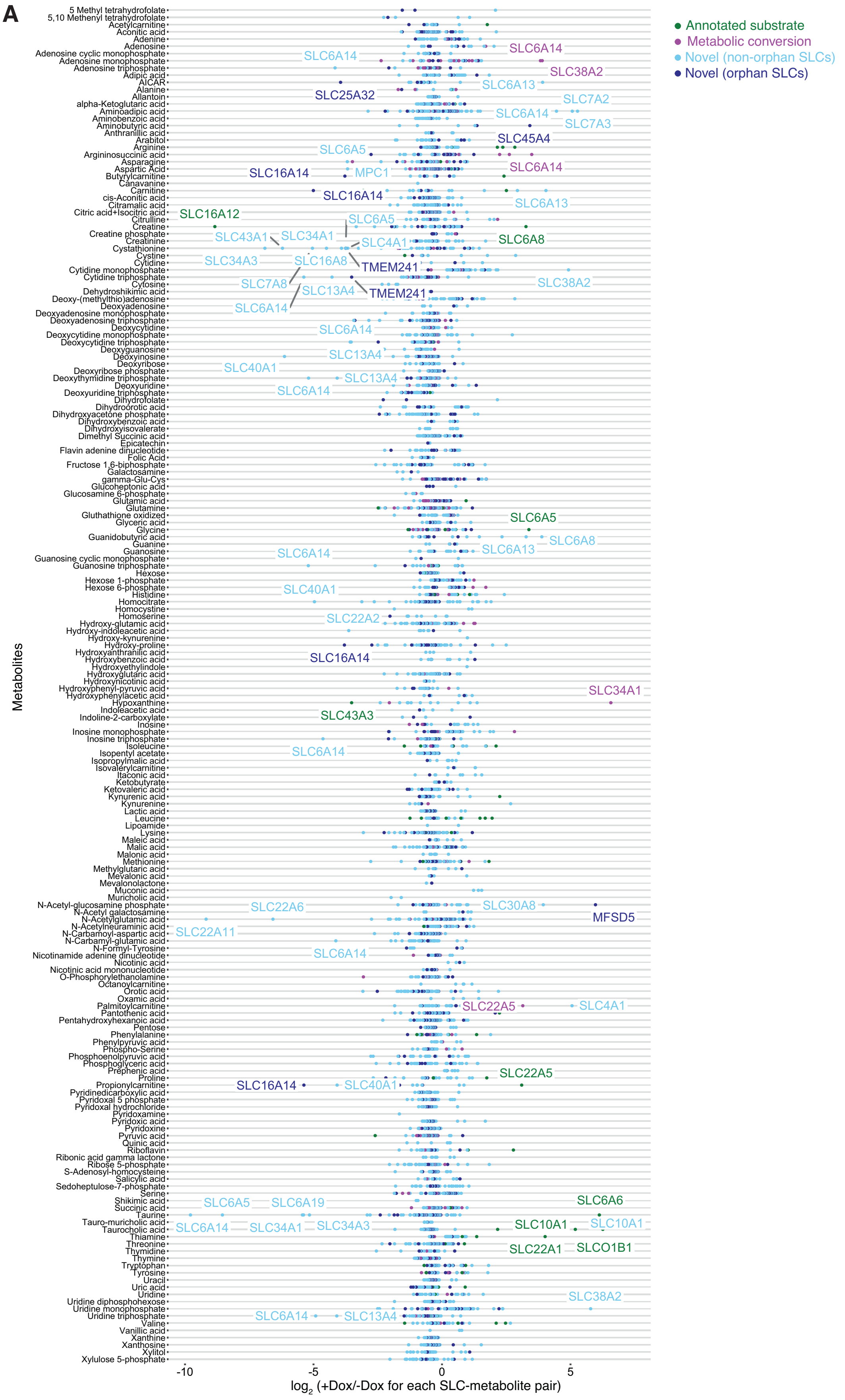
A
Annotated substrate
Metabolic conversion
Novel (non-orphan SLCs)
Novel (orphan SLCs)
Metabolites
log2 (+Dox/-Dox for each SLC-metabolite pair)
-10
-5
0
5
5 Methyl tetrahydrofolate
5,10 Methenyl tetrahydrofolate
Acetylcarnitine
Aconitic acid
Adenine
Adenosine
Adenosine cyclic monophosphate
Adenosine monophosphate
Adenosine triphosphate
Adipic acid
AICAR
Alanine
Allantoin
alpha-Ketoglutaric acid
Aminoadipic acid
Aminobenzoic acid
Aminobutyric acid
Anthranillic acid
Arabitol
Arginine
Argininosuccinic acid
Asparagine
Aspartic Acid
Butyrylcarnitine
Canavanine
Carnitine
cis-Aconitic acid
Citramalic acid
Citric acid+Isocitric acid
Citrulline
Creatine
Creatine phosphate
Creatinine
Cystathionine
Cystine
Cytidine
Cytidine monophosphate
Cytidine triphosphate
Cytosine
Dehydroshikimic acid
Deoxy-(methylthio)adenosine
Deoxyadenosine
Deoxyadenosine monophosphate
Deoxyadenosine triphosphate
Deoxycytidine
Deoxycytidine monophosphate
Deoxycytidine triphosphate
Deoxyguanosine
Deoxyinosine
Deoxyribose
Deoxyribose phosphate
Deoxythymidine triphosphate
Deoxyuridine
Deoxyuridine triphosphate
Dihydrofolate
Dihydroorotic acid
Dihydroxyacetone phosphate
Dihydroxybenzoic acid
Dihydroxyisovalerate
Dimethyl Succinic acid
Epicatechin
Flavin adenine dinucleotide
Folic Acid
Fructose 1,6-biphosphate
Galactosamine
gamma-Glu-Cys
Glucoheptonic acid
Glucosamine 6-phosphate
Glutamic acid
Glutamine
Gluthathione oxidized
Glyceric acid
Glycine
Guanidobutyric acid
Guanine
Guanosine
Guanosine cyclic monophosphate
Guanosine triphosphate
Hexose
Hexose 1-phosphate
Hexose 6-phosphate
Histidine
Homocitrate
Homocystine
Homoserine
Hydroxy-glutamic acid
Hydroxy-indoleacetic acid
Hydroxy-kynurenine
Hydroxy-proline
Hydroxyanthranilic acid
Hydroxybenzoic acid
Hydroxyethylindole
Hydroxyglutaric acid
Hydroxynicotinic acid
Hydroxyphenyl-pyruvic acid
Hydroxyphenylacetic acid
Hypoxanthine
Indoleacetic acid
Indoline-2-carboxylate
Inosine
Inosine monophosphate
Inosine triphosphate
Isoleucine
Isopentyl acetate
Isopropylmalic acid
Isovalerylcarnitine
Itaconic acid
Ketobutyrate
Ketovaleric acid
Kynurenic acid
Kynurenine
Lactic acid
Leucine
Lipoamide
Lysine
Maleic acid
Malic acid
Malonic acid
Methionine
Methylglutaric acid
Mevalonic acid
Mevalonolactone
Muconic acid
Muricholic acid
N-Acetyl-glucosamine phosphate
N-Acetyl galactosamine
N-Acetylglutamic acid
N-Acetylneuraminic acid
N-Carbamoyl-aspartic acid
N-Carbamyl-glutamic acid
N-Formyl-Tyrosine
Nicotinamide adenine dinucleotide
Nicotinic acid
Nicotinic acid mononucleotide
O-Phosphorylethanolamine
Octanoylcarnitine
Orotic acid
Oxamic acid
Palmitoylcarnitine
Pantothenic acid
Pentahydroxyhexanoic acid
Pentose
Phenylalanine
Phenylpyruvic acid
Phospho-Serine
Phosphoenolpyruvic acid
Phosphoglyceric acid
Prephenic acid
Proline
Propionylcarnitine
Pyridinedicarboxylic acid
Pyridoxal 5 phosphate
Pyridoxal hydrochloride
Pyridoxamine
Pyridoxic acid
Pyridoxine
Pyruvic acid
Quinic acid
Riboflavin
Ribonic acid gamma lactone
Ribose 5-phosphate
S-Adenosyl-homocysteine
Salicylic acid
Sedoheptulose-7-phosphate
Serine
Shikimic acid
Succinic acid
Taurine
Tauro-muricholic acid
Taurocholic acid
Thiamine
Threonine
Thymidine
Thymine
Tryptophan
Tyrosine
Uracil
Uric acid
Uridine
Uridine diphosphohexose
Uridine monophosphate
Uridine triphosphate
Valine
Vanillic acid
Xanthine
Xanthosine
Xylitol
Xylulose 5-phosphate
SLC6A14
SLC6A14
SLC38A2
SLC6A13
SLC25A32
SLC7A2
SLC6A14
SLC7A3
SLC45A4
SLC6A5
SLC6A14
SLC16A14
MPC1
SLC16A14
SLC6A13
SLC16A12
SLC6A5
SLC43A1
SLC34A1
SLC4A1
SLC6A8
SLC34A3
SLC16A8
TMEM241
SLC7A8
SLC38A2
SLC13A4
TMEM241
SLC6A14
SLC6A14
SLC13A4
SLC40A1
SLC13A4
SLC6A14
SLC6A5
SLC6A8
SLC6A14
SLC6A13
SLC40A1
SLC22A2
SLC16A14
SLC34A1
SLC43A3
SLC6A14
SLC22A6
SLC30A8
MFSD5
SLC22A11
SLC6A14
SLC22A5
SLC4A1
SLC22A5
SLC16A14
SLC40A1
SLC6A6
SLC6A5
SLC6A19
SLC10A1
SLC10A1
SLC6A14
SLC34A1
SLC34A3
SLC22A1
SLCO1B1
SLC38A2
SLC6A14
SLC13A4

 © The Author(s)

◀ **Figure EV2. Overview of SLC-metabolite pairs by category and per metabolite.**

(**A**) Visualization of all significant SLC-metabolite pairs ($P_{adj.}$ <0.05) in a per-metabolite view with metabolites on the *y* axis and $\log_2$ fold change +/−doxycycline (Dox) for each SLC-metabolite pair on the *x* axis for the combined metabolomics data sets. Colors indicate the SLC-metabolite pair category ('annotated substrate', 'metabolic conversion', 'novel (non-orphan SLCs)', 'novel (orphan SLCs)'.

© The Author(s)

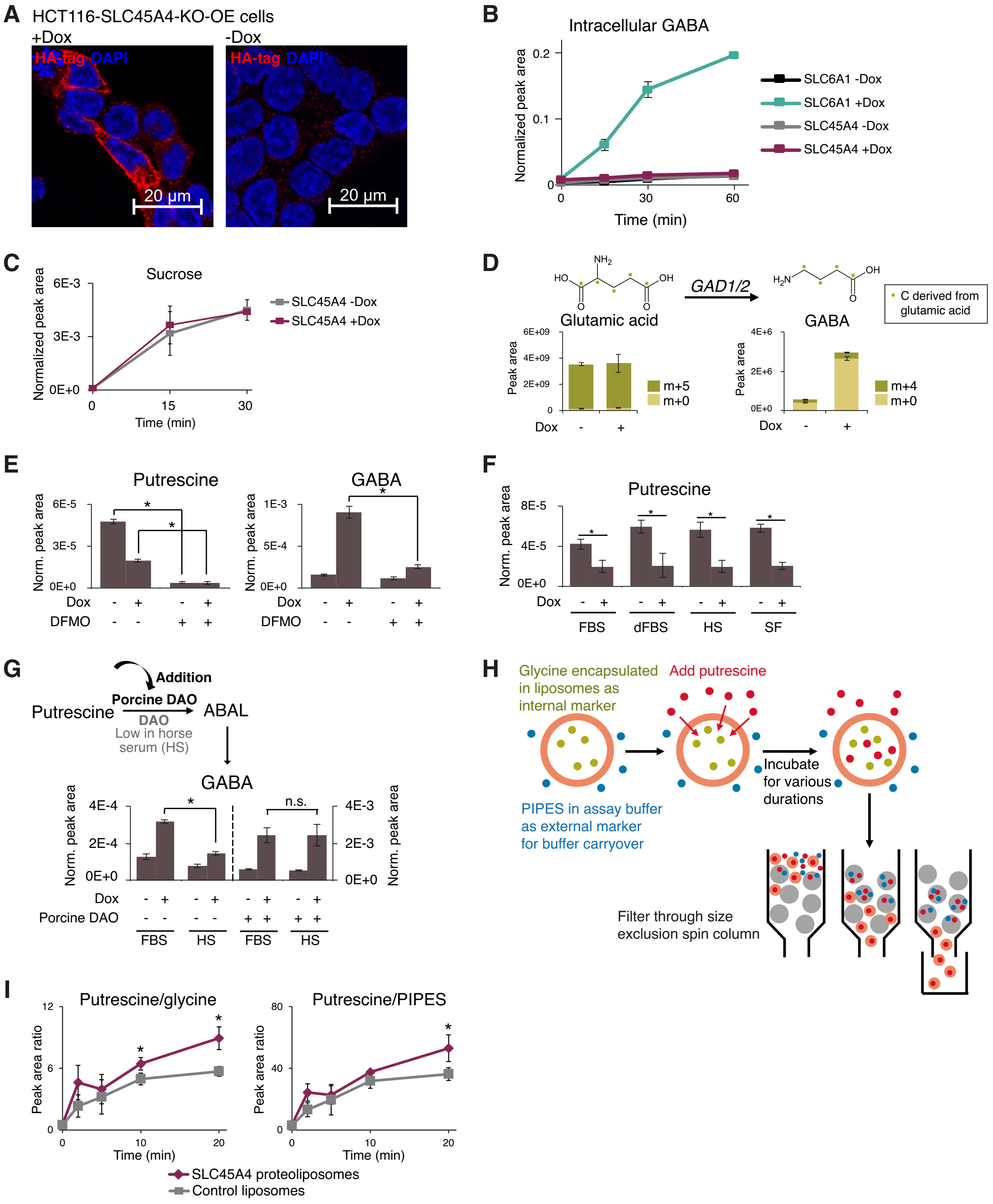
A
HCT116-SLC45A4-KO-OE cells
+Dox
-Dox
HA-tag DAPI
20 µm
B
Intracellular GABA
Normalized peak area
Time (min)
SLC6A1 -Dox
SLC6A1 +Dox
SLC45A4 -Dox
SLC45A4 +Dox
C
Sucrose
Normalized peak area
Time (min)
SLC45A4 -Dox
SLC45A4 +Dox
D
Glutamic acid
GAD1/2
GABA
C derived from glutamic acid
Peak area
m+5
m+0
m+4
Dox
E
Putrescine
GABA
Norm. peak area
Dox
DFMO
F
Putrescine
FBS
dFBS
HS
SF
G
Addition
Putrescine
Porcine DAO
DAO
Low in horse serum (HS)
ABAL
GABA
n.s.
Porcine DAO
H
Glycine encapsulated in liposomes as internal marker
Add putrescine
PIPES in assay buffer as external marker for buffer carryover
Incubate for various durations
Filter through size exclusion spin column
I
Putrescine/glycine
Putrescine/PIPES
Peak area ratio
Time (min)
SLC45A4 proteoliposomes
Control liposomes

 © The Author(s)

Figure EV3. SLC45A4 mediates GABA production via putrescine export and oxidation.

(**A**) Representative immunofluorescence images of HCT116-SLC45A4-KO-OE cells +/− 24 h doxycycline (Dox) induction. Red and blue channels show HA-tagged SLC45A4 and DAPI nuclear counterstain, respectively. (**B**) Intracellular GABA levels in HCT 116 Renilla KO cells transduced with Dox-inducible SLC6A1 overexpression construct and HCT116-SLC45A4-KO-OE cells, following +/− 24 h Dox induction in OptiMEM media +dFBS and switch to OptiMEM +dFBS +100 µM GABA for the indicated durations. Data points represent means, error bars represent s.d. ($n = 3$). (**C**) Intracellular sucrose levels in HCT116-SLC45A4-KO-OE cells following +/− 24 h Dox induction and switch to media supplemented with 1 mM sucrose for the indicated durations. Data points represent means, error bars represent s.d. ($n = 3$). (**D**) Schematic representation of glutamic acid conversion to GABA, and labeled and unlabeled abundances of intracellular glutamic acid vs GABA in HCT116-SLC45A4-KO-OE cells +/− 24 h Dox induction. Carbon atoms potentially derived from glutamic acid (and hence labeled by ^{13}C-glutamic acid) are marked by small green dots. Labeled species are as indicated for each metabolite. Bar heights represent means, error bars represent s.d. ($n = 6$). (**E**) Intracellular putrescine and GABA levels in HCT116-SLC45A4-KO-OE cells with and without ODC1 inhibition by 1 mM difluoromethylornithine (DFMO) +/− 9 h Dox induction. Bar heights represent means, error bars represent s.d. ($n = 3$). P values (Welch's t test): putrescine uninduced control vs DFMO, 1.50E-06; putrescine Dox control vs DFMO, 3.14E-05; GABA Dox control vs DFMO, 1.37E-04. (**F**) Intracellular putrescine levels in HCT116-SLC45A4-KO-OE cells cultured in regular FBS-containing media, media with dialyzed FBS (dFBS), media with horse serum (HS), or serum-free media (SF) +/− 9 h Dox induction. Bar heights represent means, error bars represent s.d. ($n = 3$). P values (Welch's t test): FBS uninduced vs Dox, 8.96E-03; dFBS uninduced vs Dox, 8.27E-03; HS uninduced vs Dox, 2.60E-03; SF uninduced vs Dox, 2.07E-04. (**G**) Intracellular GABA levels in HCT116-SLC45A4-KO-OE cells cultured in regular FBS-containing media vs horse serum (HS)-containing media, without (left) or with (right) porcine diamine oxidase (DAO) supplementation +/− 9 h Dox induction. Bar heights represent means, error bars represent s.d. ($n = 3$). P values (Welch's t test): FBS Dox vs HS Dox without DAO supplementation, 1.67E-05; FBS Dox vs HS Dox with DAO supplementation, 0.988. (**H**) Schematic of cell-free putrescine uptake assay. Glycine is encapsulated within liposomes inserted with SLC45A4 protein or protein-free control liposomes as an internal marker of liposome abundance. Liposomes are resuspended in assay buffer containing PIPES as an external marker of buffer carryover. Following addition of 100 µM putrescine and incubation for defined time points, liposome suspensions are filtered through Sephadex G-50 size exclusion chromatography (SEC) spin columns, which traps assay buffer components while allowing liposomes to flow through. The liposome-enriched eluates are then analyzed by LC–MS. (**I**) Putrescine/glycine and putrescine/PIPES peak ratios in SEC eluates of SLC45A4 proteoliposomes vs control liposomes over different uptake durations. Data points represent means, error bars represent s.d. ($n = 3$). P values (Welch's t test) of putrescine/glycine, SLC45A4 proteoliposomes vs control liposomes: $t = 0$, 0.907; $t = 2$, 0.119; $t = 5$, 0.583; $t = 10$, 0.0381; $t = 20$, 0.00960. P values (Welch's t test) of putrescine/PIPES, SLC45A4 proteoliposomes vs control liposomes: $t = 0$, 0.711; $t = 2$, 0.0556; $t = 5$, 0.669; $t = 10$, 0.110; $t = 20$, 0.0391. Where presented, asterisks (*) and n.s. on significance bars indicate $P < 0.05$ and $P \geq 0.05$, respectively. For panels (**B**, **C**, **E**, **F** and **G**), metabolite peak areas were normalized to internal standard compound peak area and further normalized to cellular protein content as described in "Methods".

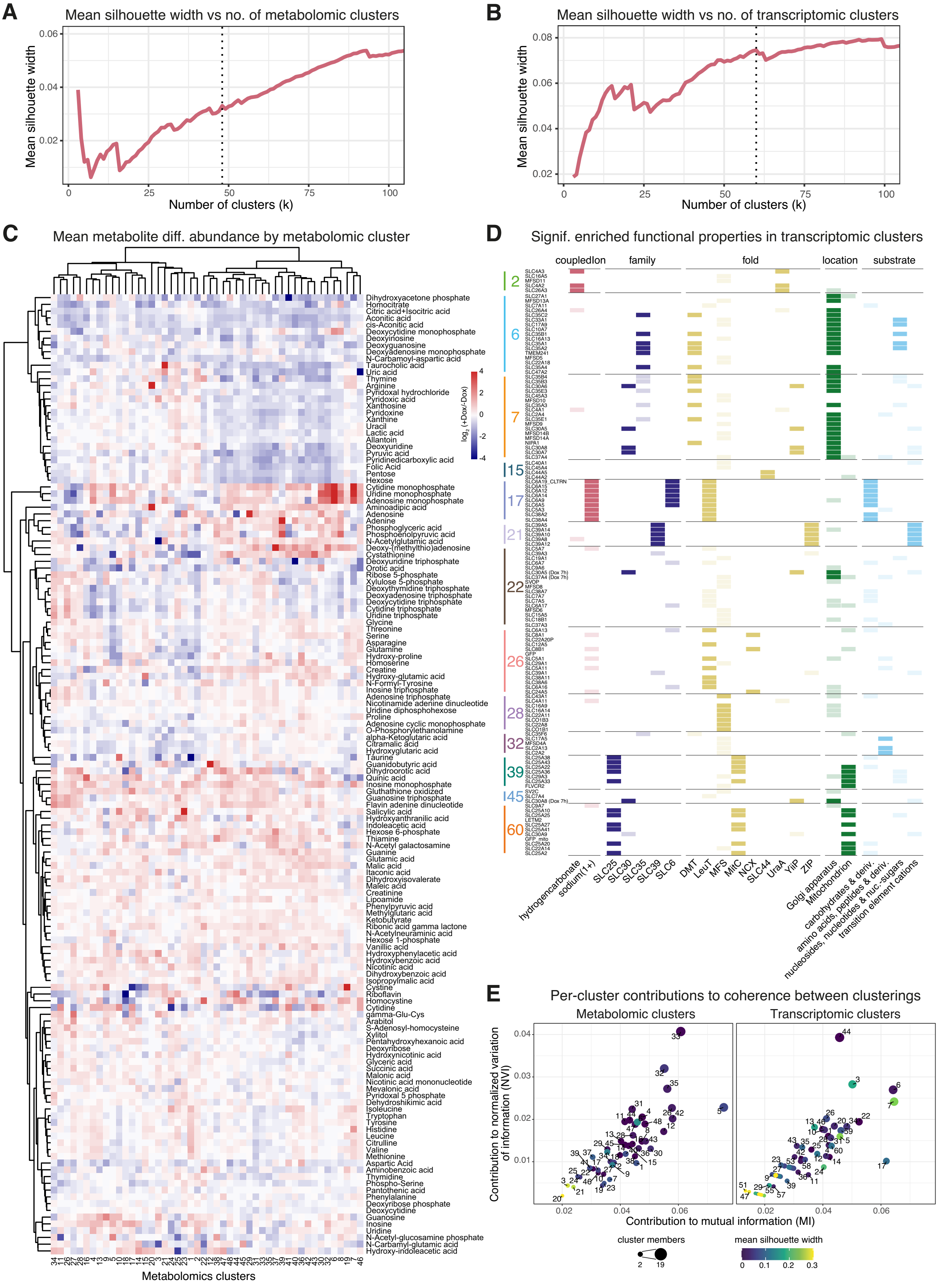
A
Mean silhouette width vs no. of metabolomic clusters
Mean silhouette width
Number of clusters (k)
B
Mean silhouette width vs no. of transcriptomic clusters
Mean silhouette width
Number of clusters (k)
C
Mean metabolite diff. abundance by metabolomic cluster
log2 (+Dox/-Dox)
Metabolomics clusters
D
Signif. enriched functional properties in transcriptomic clusters
coupledIon
family
fold
location
substrate
hydrogencarbonate
sodium(1+)
SLC25
SLC30
SLC35
SLC39
SLC6
DMT
LeuT
MFS
MitC
NCX
SLC44
UraA
YiiP
ZIP
Golgi apparatus
Mitochondrion
carbohydrates & deriv.
amino acids, peptides & deriv.
nucleosides, nucleotides & nuc.-sugars
transition element cations
E
Per-cluster contributions to coherence between clusterings
Metabolomic clusters
Transcriptomic clusters
Contribution to normalized variation of information (NVI)
Contribution to mutual information (MI)
cluster members
mean silhouette width

 © The Author(s)

Figure EV4. Analyses of clustering results identify predominant metabolite changes in metabolomic clusters and reveals enriched functional properties in transcriptomic clusters.

(**A**) Mean silhouette width across all metabolomic clusters at different cluster numbers (k = 2–100). The dashed line indicates the chosen k = 48. (**B**) Mean silhouette width across all transcriptomic clusters at different cluster numbers (k = 2–100). The dashed line indicates the chosen k = 60. (**C**) Heatmap of all clusters and metabolites based on the respective averaged log_2 fold changes per cluster. Cluster numbers are indicated on *x* axis. Metabolites are indicated on *y* axis. (**D**) Transcriptomic clusters with significant SLC functional feature enrichment (Fisher's test $P < 0.2$). (**E**) Contributions of individual clusters to normalized variation of information and mutual information between metabolomics and transcriptomics clustering. Cluster size and discreteness (as quantified by silhouette width) are additionally indicated by size and color, respectively.

© The Author(s)

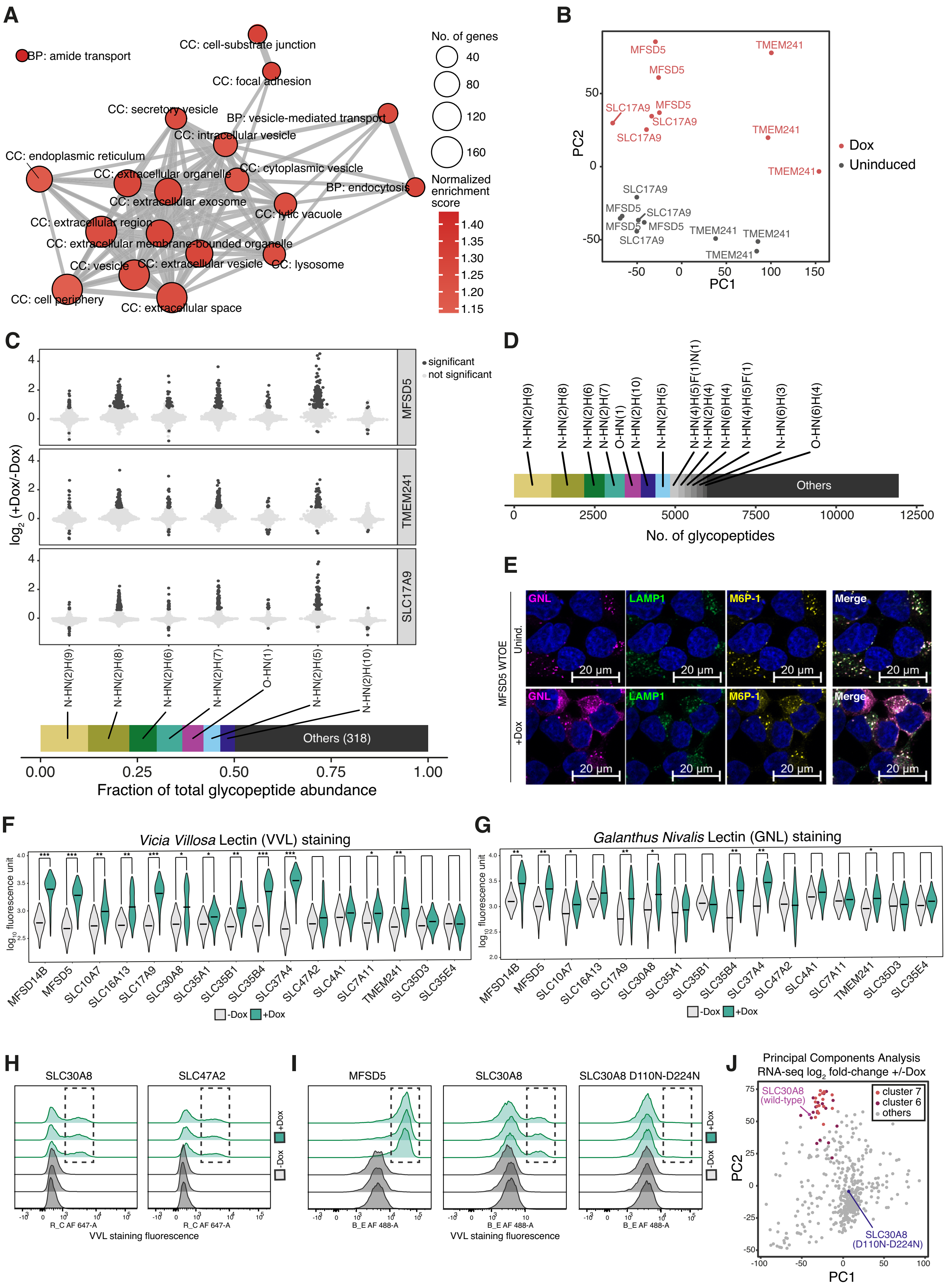
A
No. of genes
40
80
120
160
Normalized enrichment score
BP: amide transport
CC: cell-substrate junction
CC: focal adhesion
CC: secretory vesicle
BP: vesicle-mediated transport
CC: intracellular vesicle
CC: endoplasmic reticulum
CC: extracellular organelle
CC: cytoplasmic vesicle
BP: endocytosis
CC: extracellular exosome
CC: lytic vacuole
CC: extracellular region
CC: extracellular membrane-bounded organelle
CC: vesicle
CC: extracellular vesicle
CC: lysosome
CC: cell periphery
CC: extracellular space
B
PC1
PC2
Dox
Uninduced
C
log2 (+Dox/-Dox)
significant
not significant
MFSD5
TMEM241
SLC17A9
Others (318)
Fraction of total glycopeptide abundance
D
Others
No. of glycopeptides
E
GNL
LAMP1
M6P-1
Merge
MFSD5 WTOE
Unind.
+Dox
20 µm
F
Vicia Villosa Lectin (VVL) staining
G
Galanthus Nivalis Lectin (GNL) staining
log10 fluorescence unit
-Dox
+Dox
H
SLC30A8
SLC47A2
VVL staining fluorescence
I
MFSD5
SLC30A8
SLC30A8 D110N-D224N
J
Principal Components Analysis
RNA-seq log2 fold-change +/-Dox
SLC30A8 (wild-type)
SLC30A8 (D110N-D224N)
cluster 7
cluster 6
others

 © The Author(s)

Figure EV5. SLC cluster alters both N-and O-linked glycosylation signatures.

(**A**) GO terms significantly enriched ($P_{adj.}$ <0.05) by gene set enrichment analysis (GSEA) on most abundant glycoproteins detected in the data set. Abundance of each glycoprotein were calculated as sum of signal intensities of corresponding glycopeptides across 18 samples (3 replicates each of 3 cell lines – MFSD5 WT-OE, TMEM241 WT-OE, SLC17A9 WT-OE – and 2 conditions – Dox-induced and uninduced). (**B**) Principal components analysis performed on normalized glycopeptide abundances of 18 glycoproteomics samples, whereby to account for possible differences in protein amount, each glycopeptide was normalized to the total abundance of the corresponding protein. (**C**) Dox-induced vs uninduced glycopeptide abundance log_2 fold changes for 7 most abundant glycan compositions across 18 samples. Individual glycopeptides are plotted as discrete points and color-coded by significance. Bottom: stacked barplot indicating fraction of total glycopeptide signal contributed by each glycan composition, showing that these 7 compositions account for over 50% of the total glycopeptide signal. (**D**) Stacked barplot indicating number of glycopeptides attributed to each glycan composition, showing that ~40.6% (4845 out of 11,938) identified glycopeptides belong to the 7 most abundant glycan compositions. (**E**) Representative immunofluorescence images of MFSD5 WT-OE uninduced and doxycycline (Dox)-induced cells showing individual and merged channels for *Galanthus Nivalis* Lectin (GNL), LAMP1 and M6P staining with DAPI nuclear counterstain. (**F**) Cell surface *Vicia Villosa* Lectin (VVL) staining of $+/-$ Dox cells for an extended panel of cell lines overlapping between transcriptomic and metabolomic clusters. Each violin plot represents flow cytometry measurements of at least 30,000 cells pooled from 3 replicate wells, and horizontal bisecting lines indicate population geometric means. Effect sizes (Cohen's *d*) between uninduced and Dox-induced populations of each cell line are indicated by annotated brackets as follows: $*d > 0.5$; $**d > 1$; $***d > 2$. The Cohen's *d* values are: MFSD14B, 1.036; MFSD5, 1.095; SLC10A7, 0.645; SLC16A13, 0.469; SLC17A9, 1.022; SLC30A8, 0.781; SLC35A1, 0.22; SLC35B1, -0.141; SLC35B4, 1.622; SLC37A4, 1.466; SLC47A2, -0.068; SLC4A1, 0.316; SLC7A11, 0.113; TMEM241, 0.856; SLC35D3, 0.122; SLC35E4, 0.312. (**G**) intracellular GNL staining of $+/-$ Dox cells for an extended panel of cell lines overlapping between transcriptomic and metabolomic clusters. Each violin plot represents flow cytometry measurements of at least 30,000 cells pooled from 3 replicate wells, and horizontal bisecting lines indicate population geometric means. Effect sizes (Cohen's *d*) between uninduced and Dox-induced populations of each cell line are indicated by annotated brackets as follows: $*d > 0.5$; $**d > 1$; $***d > 2$. The Cohen's d values are: MFSD14B, 2.106; MFSD5, 2.437; SLC10A7, 1.001; SLC16A13, 1.287; SLC17A9, 2.19; SLC30A8, 0.915; SLC35A1, 0.614; SLC35B1, 1.464; SLC35B4, 2.244; SLC37A4, 3.125; SLC47A2, 0.436; SLC4A1, 0.331; SLC7A11, 0.75; TMEM241, 1.336; SLC35D3, 0.386; SLC35E4, -0.025. (**H**) Flow cytometry histograms comparing cell surface VVL staining of SLC30A8 and SLC47A2 WT-OE $+/-$ Dox cells. Dashed boxes indicate $+$Dox cell populations with increased VVL staining. (**I**) Flow cytometry histograms comparing cell surface VVL staining of MFSD5, SLC30A8 wild-type, and SLC30A8 D110N-D224N (transport-deficient mutant) $+/-$ Dox cells. Dashed boxes indicate $+$Dox cell populations with increased VVL staining, which is absent in the transport-deficient mutant. (**J**) Principal components analysis of gene expression log_2 fold change profiles of SLC30A8 D110N-D224N along with the 450 SLCs used in transcriptomic clustering.

© The Author(s)
